# Quantifying Workload and Injury Risk Among Cricket Fast Bowlers: A Systematic Review and Meta-Analysis

**DOI:** 10.7759/cureus.104847

**Published:** 2026-03-08

**Authors:** Arun Chelladurai, Sai Aditya Raman, Hari Kirthen, Arya Nandana, Gayathri Thiruvengadam, Nimishaanth S. S., Thiagarajan K. A., Arumugam Sivaraman

**Affiliations:** 1 Sports and Exercise Sciences, Sri Ramachandra Institute of Higher Education and Research, Chennai, IND; 2 Arthroscopy and Sports Medicine, Sri Ramachandra Institute of Higher Education and Research, Chennai, IND; 3 Allied Health Sciences, Sri Ramachandra Institute of Higher Education and Research, Chennai, IND; 4 Sports and Exercise Science, Sri Ramachandra Institute of Higher Education and Research, Chennai, IND

**Keywords:** acute-to-chronic workload ratio, cricket-related injuries, fast bowling, indian cricketers, workload management

## Abstract

Bowling workload is a well-recognised contributor to gradual-onset injuries in cricket fast bowlers; however, considerable variability exists in how workload is defined, quantified, and interpreted across studies. Although the acute:chronic workload ratio (ACWR) is widely applied in cricket research, its use remains debated regarding its predictive validity, mathematical limitations, and inconsistent methodological application. This systematic review aimed to synthesise existing evidence on workload quantification and workload-injury associations in cricket fast bowlers and to evaluate acute-chronic workload exposure using a restricted meta-analytic approach. A comprehensive search of MEDLINE, Embase, SPORTDiscus, CINAHL, Scopus, Web of Science, PubMed, and Google Scholar was conducted, spanning 2000 to 2024. Boolean search combinations included terms such as "cricket," "fast bowler," "workload," "bowling load," "acute:chronic workload ratio," "injury," and "injury risk." Study screening was performed using Rayyan (Rayyan Systems Inc., Cambridge, MA, USA) following duplicate removal in Mendeley (Elsevier B.V., Amsterdam, Netherlands), and eligible studies were independently assessed by three reviewers. Thirty-three studies met the inclusion criteria and were synthesised narratively, while six prospective cohort studies reporting acute-chronic workload exposure with extractable effect estimates were included in a random-effects meta-analysis. The included studies involved elite, sub-elite, and junior male and female fast bowlers and employed diverse workload monitoring methods, including manual tracking, wearable technology, subjective measures, and biomechanical assessments. Across studies, high acute workloads, rapid workload escalations, and inadequate recovery were consistently associated with increased injury risk, particularly involving the lumbar spine. These associations were broadly consistent across competitive levels, although elite cohorts demonstrated more robust monitoring protocols and junior populations exhibited greater vulnerability during rapid load escalation. Elevated ACWR values, typically ranging from approximately 1.4 to 2.0 and above, were associated with higher injury incidence, whereas greater chronic workload exposure appeared protective. Meta-analysis demonstrated a significant association between elevated acute-chronic workload exposure and injury risk (RR = 2.45; 95% CI: 1.48-4.06), although substantial heterogeneity was observed (I^2^ ≈ 77%), likely attributable to variability in workload metrics, inconsistency in injury definitions, and differences in study populations. These findings indicate that acute workload spikes relative to recent chronic workload are consistently linked to increased injury risk in cricket fast bowlers; however, heterogeneity and study-level bias limit definitive causal inference. Consequently, workload monitoring strategies should emphasise progressive load development, adequate recovery, and individualised interpretation rather than reliance on rigid threshold values. Notably, evidence specific to Indian fast bowlers remains limited, underscoring a critical gap for future research.

## Introduction and background

Cricket is played across multiple formats, including Test (five-day matches), One-Day International (ODI; 50 overs per team), and Twenty20 (T20; 20 overs per team), each imposing varied technical, tactical, and physiological demands on players. Fast bowlers, whose primary role is to deliver the ball at maximum velocity toward the opposing batsman in an attempt to take wickets, are responsible for a substantial proportion of a team's attacking play and experience the highest biomechanical and physiological loads due to repetitive high-intensity efforts [1,2]. Bowling at velocities frequently exceeding 140 km·h^-1^ demands a long run-up, explosive trunk rotation, and substantial lower-limb force production, exposing the lumbar spine, shoulders, and lower limbs to considerable mechanical stress and increasing the risk of overuse injuries, particularly lumbar stress fractures (LSFs) [3,4]. Bowling action technique - classified as side-on, front-on, semi-open, or mixed - also influences load distribution, with mixed actions associated with excessive trunk rotation and a greater risk of lumbar spine injury [5,6].

Workload is one of the most modifiable risk factors for injury in cricket fast bowlers and is commonly defined as the cumulative physical stress imposed during training and competition. This includes both external workload (e.g., number of deliveries bowled, distance covered) and internal workload (e.g., physiological strain or perceived exertion) [7-9]. Compared with other playing roles, elite fast bowlers perform up to 80% greater total running distance, two to seven times more high-intensity efforts, and experience reduced recovery between efforts [10,11]. Consequently, fast bowlers sustain the greatest injury burden in cricket, missing approximately 16% of playing time compared with less than 5% in other roles, with match bowling presenting the highest injury risk, particularly for the lumbar spine [12,13].

Evidence suggests that both excessive and insufficient workloads are associated with increased injury risk, supporting a non-linear workload-injury relationship [14]. Weekly bowling volumes exceeding 188 deliveries with fewer than two rest days, or volumes below 123 deliveries with extended recovery periods, have been associated with greater injury risk compared with moderate workloads (123-188 deliveries per week with three to four days of recovery) [12,14]. Acute workload spikes are particularly hazardous; bowling volumes exceeding 234 deliveries within seven days have been associated with a markedly increased incidence of LSFs, while bone stress injuries may occur when high short-term workloads are imposed on a background of low chronic workload exposure [15-18].

The acute:chronic workload ratio (ACWR) is a widely adopted model for managing these risks, with an optimal “sweet spot” of 0.8-1.3 associated with performance gains and reduced injury incidence [19,20]. Ratios exceeding approximately 1.4-2.0 have been associated with increased injury risk in fast bowlers, particularly when acute workload increases are not supported by adequate chronic load development [7,8]. However, the interpretation of ACWR remains contentious, with limited cricket-specific validation and substantial variation in calculation methods, exposure windows, and workload metrics [2]. Moreover, many studies rely primarily on external workload measures, such as delivery counts, without adequately accounting for intensity differences between training and match play.

It is important to note that the ACWR model has been subject to considerable methodological scrutiny in recent years. Concerns have been raised regarding its predictive validity, mathematical coupling artefacts inherent in the coupled ACWR formula, sensitivity to parameter choices (e.g., acute and chronic time windows), and the risk of oversimplifying complex, multifactorial injury mechanisms into a single ratio. Despite these limitations, the ACWR remains the most widely reported workload metric in cricket research and thus forms a central focus of this review, while acknowledging these ongoing debates.

Despite advances in workload-injury research, important gaps remain. Most evidence derives from Australian and English cohorts, with limited data specific to Indian fast bowlers, who are exposed to distinct contextual demands including subcontinental pitch characteristics, congested competition schedules (e.g., the Indian Premier League), and heterogeneous anthropometric and developmental profiles [21]. In addition, workload quantification methods lack standardisation, and the relationship between workload exposure and injury patterns in Indian fast bowlers remains poorly understood.

Therefore, the purpose of this systematic review was to synthesise existing evidence on bowling workload and injury risk in cricket fast bowlers. Specifically, the review aimed to (i) examine methods used to quantify bowling workload, (ii) evaluate associations between workload exposure and injury incidence, (iii) assess evidence relating to acute-chronic workload models, and (iv) identify evidence gaps relevant to Indian fast bowlers. By consolidating current findings and highlighting methodological limitations, this review seeks to inform evidence-based workload management strategies and guide future research in cricket fast bowling.

## Review

Methods

**Figure 1:**
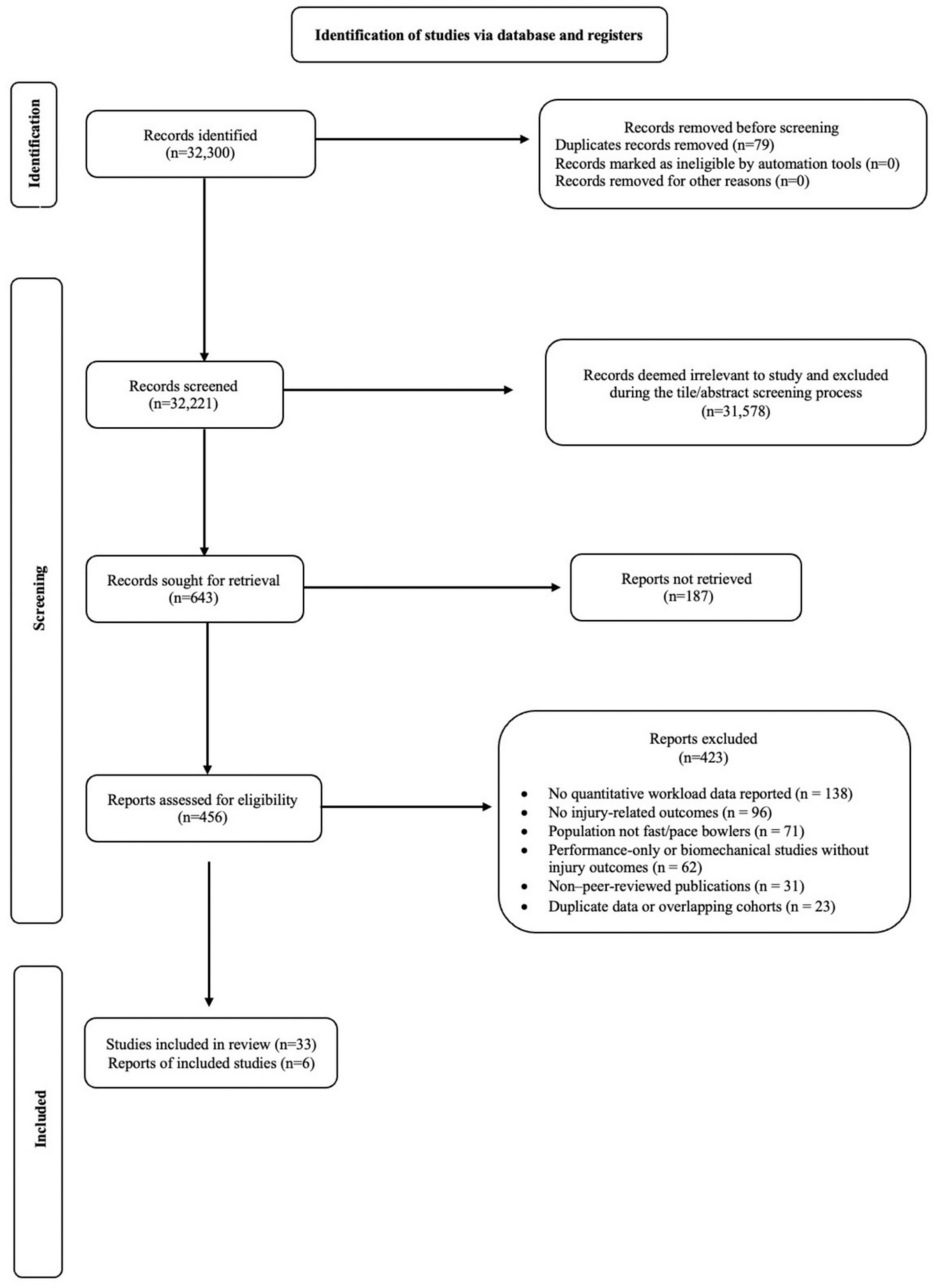
PRISMA 2020 flow diagram illustrating the study identification, screening, eligibility, and inclusion process Preferred Reporting Items for Systematic Reviews and Meta-Analyses (PRISMA) [22] flow diagram summarises the systematic study selection process for the review examining workload monitoring and injury risk in cricket fast bowlers.

This systematic review process was conducted and reported in accordance with the Preferred Reporting Items for Systematic Reviews and Meta-Analyses (PRISMA) 2020 guidelines [22], ensuring transparency and methodological rigour in the identification, screening, and inclusion of studies, with the study selection process summarised using a PRISMA flow diagram (Figure 1). As the review involved the synthesis of previously published literature, no new human or animal participants were recruited, and informed consent was not required. Institutional approval for conduct and submission was obtained from the Institutional Ethics Committee of Sri Ramachandra Institute of Higher Education and Research (SRIHER), Chennai, India, and the manuscript was approved by the Publications Guidelines and Monitoring Committee (PGMC), SRIHER (PGMC Ref. No.: SRIHER2025/PGMC612/CSS).

Studies were eligible for inclusion if they involved male and/or female cricket fast (pace) bowlers at any competitive level and reported quantitative measures of bowling workload, including frequency, volume, intensity, or time/duration. Eligible studies examined associations between workload exposure and injury outcomes, such as injury incidence, prevalence, or workload-related risk factors, and included extrinsic factors influencing workload or injury risk, such as biomechanics or training variables. Studies employing objective workload assessment approaches, including manual databases (e.g., match scorecards or injury surveillance systems) or technological tools (e.g., global positioning systems (GPS), wearable sensors, inertial measurement units (IMUs), video analysis, or force platforms), were considered. Only full-text, peer-reviewed journal articles published in English were included. Studies were excluded if they were conference abstracts, editorials, non-peer-reviewed publications, review articles of any type, or if they did not involve cricket fast bowlers, lacked objective workload quantification, or focused exclusively on performance, coaching, or internal workload measures without reference to injury-related outcomes.

A comprehensive literature search was conducted across multiple electronic databases, including MEDLINE, Embase, SPORTDiscus, CINAHL, Scopus, Web of Science, PubMed, and Google Scholar. The search covered the period from January 2000 to December 2024, encompassing 24 years of published research. The search was last updated on 20 December 2024. Boolean search combinations were constructed using the following terms: (“cricket” OR “cricket fast bowler” OR “pace bowler”) AND (“workload” OR “bowling load” OR “training load” OR “match load” OR “acute:chronic workload ratio” OR “ACWR”) AND (“injury” OR “injury risk” OR “overuse injury” OR “stress fracture” OR “lumbar injury”). Additional terms related to monitoring technologies (e.g., “GPS,” “accelerometer,” “inertial measurement unit,” “wearable sensor”) and biomechanics (e.g., “bowling biomechanics,” “spinal loading”) were included in supplementary searches. Search strategies were adapted for each database to accommodate differences in indexing and controlled vocabulary.

All retrieved citations were exported to Mendeley Reference Manager (Elsevier B.V., Amsterdam, Netherlands), where duplicate records were automatically removed prior to screening. Title and abstract screening were performed using Rayyan systematic review software (Rayyan Systems Inc., Cambridge, MA, USA).

Three reviewers independently screened titles and abstracts for eligibility, followed by full-text assessment of potentially relevant studies. Any disagreements were resolved through discussion, with final arbitration by the lead reviewer, who also oversaw the final study inclusion and synthesis process. Inter-reviewer agreement during the full-text screening stage was assessed using Cohen’s kappa statistic, yielding a kappa value of 0.87, indicating strong agreement among the three reviewers.

Data were extracted using a predefined extraction form and included study characteristics (authors, year, design), participant characteristics (sample size, competitive level, age, and sex), bowling workload metrics (frequency, volume, intensity, duration, and composite indices such as the ACWR), workload assessment tools and technologies, and reported injury-related outcomes, including injury incidence, prevalence, anatomical location, and workload-related risk estimates. These data were synthesised narratively, with a restricted meta-analysis performed for studies reporting comparable acute-chronic workload exposure and extractable effect estimates.

All statistical analyses for the meta-analysis were conducted using R (version 4.3.2; R Foundation for Statistical Computing, Vienna, Austria) with the metafor package (version 4.4-0). Random-effects models were fitted using restricted maximum likelihood (REML) estimation. Heterogeneity was quantified using the I^2^ statistic, τ^2^, H^2^, and Cochran’s Q test. Forest and funnel plots were generated using the same software. Effect sizes were extracted as relative risks (RRs) with 95% confidence intervals (CIs) from each study; where studies reported odds ratios (ORs), these were converted to RRs using standard formulae applicable to the baseline incidence reported in each study.

A leave-one-out sensitivity analysis was planned a priori to assess the influence of individual studies on the pooled estimate. Formal subgroup analyses by competitive level (elite versus junior/sub-elite) and workload metric type (external versus internal) were not performed due to the small number of studies included in the meta-analysis (n = 6), which limited statistical power for meaningful subgroup comparisons. These limitations are acknowledged in the Discussion section.

Table 1 summarises the studies examining bowling workload, injury risk, and workload monitoring in cricket fast bowlers. The included studies focused on (i) bowling workload characteristics, including frequency, intensity, and volume; (ii) injury incidence and prevalence in relation to bowling workload exposure; and (iii) monitoring of bowling workload using technology-based tools such as GPS, accelerometers, IMUs, and video-based analysis.

**Table 1:** Characteristics and key findings of studies examining bowling workload, injury risk, and workload monitoring in cricket fast bowlers The included studies investigated bowling workload parameters (frequency, intensity, and volume), injury incidence and prevalence in relation to workload exposure, and workload monitoring using technology-based and subjective measures (e.g., GPS, IMUs, accelerometers, video analysis, and sRPE). Information is provided on study design, participant characteristics, workload measurement approaches, study aims, analysed risk factors, and key outcomes. Evidence spans junior, sub-elite, elite, and professional male and female fast bowlers across multiple competitive levels. Overall, the findings demonstrate that excessive acute workloads, rapid workload changes, insufficient recovery, and age-related vulnerability, particularly in younger fast bowlers, are consistently associated with increased injury risk, especially involving the lumbar spine, while appropriately managed chronic workloads appear protective. ACWR: acute:chronic workload ratio; AMS: athlete management system; CMJ: countermovement jump; EWMA: exponentially weighted moving average; GPS: global positioning system; IMU: inertial measurement unit; LBSI: lumbar bone stress injury; LSF: lumbar stress fracture; RR: relative risk; sRPE: session rating of perceived exertion

Author	Study Design	Participants	Technologies/Measures	Study Focus
Hulin et al., 2014 [7]	Prospective cohort study	28 elite fast bowlers, 43 individual seasons	External workload: weekly ball counts; Internal workload: sRPE; ACWR calculation	Determine whether spikes in acute workload relative to chronic workload are associated with increased injury risk in fast bowlers
Dennis et al., 2003 [14]	Prospective cohort study	90 elite male fast bowlers (mean age 27 years)	Match scorecards, Training logs, Cricket Australia Injury Surveillance	Assess how fast bowling workload patterns relate to specific injury types
Orchard et al., 2015 [16]	Prospective cohort risk factor study	235 elite fast bowlers, observed over 14,600 player innings across 15 years	Injury surveillance database; manual/statistical match workload logs	Documenting overs bowled and injury type patterns (acute, medium-term, seasonal, and career)
Alway et al., 2019 [17]	Prospective cohort epidemiological study	368 professional English County fast bowlers (2010-2016)	ECB Injury Surveillance System, Match workload records, Logistic regression	Incidence and seasonal variation of lumbar stress fractures and their association with bowling workload
Orchard et al., 2015 [18]	Prospective cohort study	235 fast bowlers monitored over 15 years	Manual/match workload logs and injury surveillance data from Cricket Australia	Examine whether high match workloads over short to medium durations are associated with increased injury risk
Dennis et al., 2005 [23]	Prospective cohort study	44 male junior fast bowlers (mean age 14.7 ± 1.4 years)	Daily self-report diary for bowling workload; Physiotherapist-confirmed injury validation	Assess whether bowling workload is a risk factor for overuse injuries in junior fast bowlers
Davies et al., 2008 [24]	Prospective observational cohort study	46 elite junior fast bowlers, aged 11-18 years	Anthropometric/postural assessments; Physical fitness screenings; Bowling technique analysis; Workload tracking	Compile injury profile and identify risk factors in elite junior fast bowlers
McNamara et al., 2013 [25]	Prospective observational study	26 elite junior cricketers (mean age 17.7 ± 1.1 years)	GPS tracking; Salivary hormone analysis; CMJ; Perceptual well-being questionnaire	Evaluate workload and fatigue responses in fast and non-fast bowlers
Blanch et al., 2015 [26]	Retrospective cohort study	215 fast bowlers from Cricket Australia 10programs, tracked over 14 years	Injury surveillance databases, match bowling exposure logs	Examine age-specific variation in incidence and severity of overuse injuries
McNamara et al., 2015 [27]	Experimental, repeated-measures design	7 nationally-level fast bowlers	MinimaxX microtechnology unit (Catapult Sports), radar gun for ball velocity	Examine between-bowler variability in PlayerLoad, ball velocity, and bowling execution
Stretch, 2015 [28]	Epidemiological, retrospective cross-sectional survey	2,081 elite schoolboy cricketers (U15, U17, U18)	Questionnaires, manual injury surveillance reporting	Determine injury incidence, type, and demographics in youth cricketers
McNamara et al., 2017 [29]	Repeated-measures, field-based	12 high-performing fast bowlers (mean age 20.3 ± 2.2 year)	Wearable microtechnology (PlayerLoad™) accelerometers, gyroscopes, radar gun	Assess whether microtechnology accurately reflects bowling intensity
Patel et al., 2017 [30]	Retrospective cohort with regression tree analysis	NZ U-19 fast bowlers (1986-2008)	Decision tree modelling, youth cricket performance datasets	Predictors of future Test cricket selection based on youth metrics
Greig and Nagy, 2017 [31]	Repeated-measures, field-based experimental	10 male academy fast bowlers (mean age 18.1 ± 0.6 years)	GPS-mounted triaxial accelerometers (100 Hz) at lumbar and cervicothoracic spine	Spinal loading during a seven-over fast-bowling spell
Webster et al., 2018 [32]	Descriptive and comparative study	19 male provincial cricketers	GPS tracking (distance, movement intensity, HR, sprints)	Compare physical demands of one-day match vs. typical training session
Warren et al., 2018 [33]	Prospective cohort study	29 male fast bowlers (aged 15-18), England & Wales Development Programme	Manual workload tracking (balls/week), injury monitoring, mixed-effects modelling	Relationship between ACWR and injury risk; individual variability
Soomro et al., 2018 [34]	Descriptive cross-sectional survey	170 male Level 2 cricket coaches	Web-based questionnaire survey	Assess physical conditioning and workload monitoring practices
Soomro et al., 2018 [35]	Prospective cohort study	408 male sub-elite cricketers	MyCricket scorebook, physio-reported injuries	Injury incidence, patterns, and risk factors in sub-elite cricket
Vickery et al., 2018 [36]	Comparative cross-sectional field study	42 male national-level and U19 cricketers	Heart rate monitors, GPS tracking, RPE scales, Video analysis	Compare physical and technical demands of training vs. match-play
Greig et al., 2019 [37]	Repeated measures, field-based	12 male academy fast bowlers (mean age 18.7 ± 0.7 years)	GPS with triaxial accelerometry	Effect of sub-maximal bowling on ball speed and lumbar loading
Cooke et al., 2019 [38]	Repeated measures observational	22 elite male cricketers (seamers and non-seamers)	Force plates (CMJ), GPS, PlayerLoad™, RPE/soreness scales	Compare neuromuscular fatigue and workload responses post-training vs. match-play
Ahmun et al., 2019 [39]	Prospective cohort during overseas tours	39 elite adolescent male international cricketers (mean age 17.5 ± 0.8 year)	sRPE for internal load; Daily wellness self-reports; Injury/illness tracking; 3-day acute and 14-day chronic workloads	Link between internal workload, wellness markers, and injury/illness risk during tours
Warren et al., 2019 [40]	Prospective cohort	90 elite female cricketers (mean age 23.4 ± 4.8 years) across 6 teams	Manual injury surveillance	Injury incidence, type, mechanism, and activity context in elite women’s T20 cricket
Gabbett et al., 2019 [41]	Prospective cohort study	28 elite male fast bowlers (mean age 26 ± 5 years)	Session-RPE; coupled vs. uncoupled ACWR calculations	Compare injury risk prediction between coupled and uncoupled ACWR methods
Garcia-Byrne et al., 2020 [42]	Retrospective observational study (2016–17)	34 elite female T20 players	GPS units (distance, PlayerLoad, velocity zones); Smartphone app (sessional RPE)	Compare internal and external workloads across competition levels in women’s T20 cricket
Tysoe et al., 2020 [43]	Prospective cohort study	49 professional male fast bowlers from six First-Class County Cricket teams	Weekly bowling volume tracking (overs)	Evaluate ‘differential load’ as a predictor of injury risk vs. ACWR
Perera and Hägglund, 2020 [44]	Prospective cohort study	90 elite Australian female cricketers (age 16-38 years)	Cricket Australia’s Athlete Management System	Injury incidence, nature, severity, and mechanisms
Walter et al., 2021 [45]	Retrospective (12-month self-reporting)	35 domestic NZ fast bowlers	Electronic questionnaire (injury type, onset, site, diagnosis)	Examine injury prevalence, type, and location
McGrath et al., 2021 [46]	Cross-sectional cohort study	Fast bowlers	Thoracic-mounted IMU (250 Hz), Machine learning (gradient boosting)	Use of IMU + ML to estimate ball release speed and perceived intensity zone
Sims et al., 2021 [47]	Retrospective cohort study (five seasons)	222 elite youth male fast bowlers (mean age 17.4 ± 1.1 years)	Cricket Australia’s AMS; Musculoskeletal screenings; Bowling volume logs; Technique assessments	Identify risk factors for lumbar bone stress injury
Hoyne et al., 2022 [48]	Cross-sectional observational cohort	26 elite cricket players (8 female, 18 male)	Field observation of throws; Self-reported logs	Validity of self-reported throwing volumes in elite cricket
Christie et al., 2024 [49]	Prospective observational cohort study	14 elite male fast bowlers (South African national team)	Manual workload logs; injury surveillance; ACWR modelling	Assess relationship between bowling volume and in-season injury risk
Pandikumar et al., 2024 [50]	Retrospective analytical study (predictive modelling)	12 elite fast bowlers (mean age ≈ 20 yrs)	Predictive modelling using workload, biomechanics, fitness and prior injury	Predicting injury risk

Risk of bias assessment

Risk of bias was assessed for studies included in the meta-analysis using the Newcastle-Ottawa Scale (NOS) for cohort studies. The NOS evaluates methodological quality across three domains: selection of cohorts (maximum 4 points), comparability of study groups (maximum 2 points), and outcome assessment (maximum 3 points), yielding a total score of up to 9 points.

All six studies included in the quantitative synthesis employed prospective cohort designs and demonstrated generally high methodological quality, with NOS scores ranging from 8 to 9. Risk-of-bias assessments were conducted independently by two reviewers, and any discrepancies in scoring were resolved through discussion until consensus was reached. Where disagreements persisted, the lead reviewer adjudicated. Across studies, workload exposure was clearly defined and measured prospectively, injury outcomes were ascertained using established surveillance or clinical reporting systems, and statistical analyses adjusted for key confounding variables such as age, playing level, prior injury history, and workload exposure. Outcome assessment was conducted independently of workload measurement, and follow-up periods were sufficient to capture gradual-onset and overuse injuries relevant to fast bowling.

Although all included studies were rated as having low overall risk of bias, limitations inherent to observational research, such as potential residual confounding and exposure misclassification, remain. Consequently, pooled effect estimates from the meta-analysis were interpreted as associative rather than causal.

A summary of the risk-of-bias assessment is presented in Table 2.

**Table 2:** Risk of bias (RoB) assessment of included studies using the Newcastle-Ottawa Scale (NOS) Studies were assessed across three domains: Selection (maximum 4 stars), Comparability (maximum 2 stars), and Outcome (maximum 3 stars), yielding a maximum total score of 9 stars. All included studies demonstrated low risk of bias, with total NOS scores ranging from 8 to 9. High scores in the Selection domain reflect appropriate cohort selection and exposure ascertainment, while consistent scoring in the Comparability domain indicates adequate control for key confounding variables. Outcome assessment scores suggest reliable outcome measurement and sufficient follow-up across studies. Stars represent the number of criteria met within each NOS domain. Overall risk of bias was categorised as low for studies scoring ≥7 stars.

Study	Selection (4)	Comparability (2)	Outcome (3)	Total (/9)	Overall RoB
Hulin et al., 2014 [7]	★★★★	★★	★★★	9	Low
Warren et al., 2018 [33]	★★★★	★★	★★★	9	Low
Ahmun et al., 2019 [39]	★★★	★★	★★★	8	Low
Gabbett et al., 2019 [41]	★★★★	★★	★★	8	Low
Tysoe et al., 2020 [43]	★★★★	★★	★★	8	Low
Christie et al., 2024 [49]	★★★★	★★	★★★	9	Low

Results

This systematic review synthesised findings from 33 studies examining the relationship between bowling workload and injury risk among cricket fast bowlers. The included studies comprised prospective cohort designs (n = 18), retrospective cohort studies (n = 6), cross-sectional studies (n = 4), repeated-measures observational or experimental designs (n = 4), and predictive or mixed-method studies (n = 1). Study populations included elite, sub-elite, and junior male and female fast bowlers, with sample sizes ranging from 4 to 408 participants. Given the heterogeneity in study designs, workload metrics, injury definitions, and statistical approaches, a narrative synthesis was undertaken for the majority of studies, with a restricted meta-analysis conducted for studies examining acute-chronic workload exposure.

Workload Quantification Methods

Studies investigating workload quantification employed both external and internal workload measures. External workload was most commonly quantified using manual methods such as match scorecards, training logs, and player diaries, particularly for tracking deliveries bowled and overs completed during match play [14,18,23,33]. While these approaches were considered reliable for match exposure, limitations were noted for training workloads due to reporting inconsistencies and delayed data entry [32].

More recent studies utilised wearable microtechnology, including GPS units, accelerometers, gyroscopes, and IMUs, to quantify PlayerLoad™, running distance, acceleration profiles, and bowling intensity [27,29,36,37,46]. These studies demonstrated strong correlations between PlayerLoad™ and perceived effort (r = 0.83) as well as ball velocity (r = 0.82), supporting the validity of microtechnology for external workload monitoring, although greater variability was observed at lower intensities (coefficient of variation, CV ≈ 19%) [29]. Lumbar spine load was found to exceed cervicothoracic load during a seven-over spell, with no acute fatigue observed; GPS accelerometry was demonstrated to be feasible for on-field monitoring [31].

Internal workload was predominantly assessed using session rating of perceived exertion (sRPE) multiplied by session duration [39,41,42]. Elevated short-term internal workloads, particularly high three-day sRPE values, were associated with a 2.5-fold increase in injury risk [39]. Several studies supplemented workload measures with wellness questionnaires assessing sleep quality, muscle soreness, stress, and mood. However, self-reported workload monitoring demonstrated reduced accuracy when reporting was delayed beyond 24 hours, with error rates exceeding 36% [48].

Workload-Injury Associations

Across studies, a consistent association was observed between high or poorly managed workloads and injury incidence, particularly involving the lumbar spine, shoulder, and lower limb. High acute workloads, defined as bowling more than 50 overs within five days, were associated with an increased risk of injury in the subsequent month (RR = 1.54) [18,38]. Several studies reported a U-shaped workload-injury relationship, whereby both excessively low and excessively high weekly bowling volumes were associated with elevated injury risk [14].

In junior fast bowlers, increased bowling frequency and reduced recovery time were strongly associated with overuse injuries [24]. Bowling with fewer than 3.5 rest days between sessions was associated with a 3.1-fold increase in injury risk, while bowling more than 2.5 days per week or exceeding 50 deliveries per day also significantly increased injury risk [23].

Injury Type and Anatomical Distribution

Injury patterns varied according to workload characteristics, age, and tissue type [45]. Lumbar spine injuries, including LSFs, were among the most prevalent and severe injuries reported. Epidemiological surveillance studies reported LSF incidence rates of 0.16 per 10,000 deliveries, with seasonal peaks observed during mid- and late-season competition periods [17]. In elite women’s T20 cricket, injury incidence was highest in the shoulder, lower back, and knee, with catching and throwing identified as major contributing activities and gradual-onset injuries being common [40]. Peak short-term workloads exceeding 234 deliveries within seven days were associated with an 11-fold increase in LSF risk, with younger fast bowlers demonstrating significantly higher susceptibility [17,44].

Tissue-specific analyses revealed that bone stress injuries were associated with high medium-term workloads combined with low career workload exposure, whereas tendon injuries were more commonly associated with high acute and previous-season workloads [16,35]. Age further influenced injury patterns, with fast bowlers under 22 years exhibiting a 3.7-6.7 times higher risk of bone-related injuries, while those over 31 years demonstrated a greater propensity for tendon injuries [26]. Intrinsic factors such as younger age, increased height, and higher bowling speeds were also identified as predictors of lumbar bone stress injury [47].

Acute:Chronic Workload Ratio (ACWR)

The ACWR emerged as a key workload metric across multiple prospective cohort studies. Elevated ACWR values were consistently associated with increased injury risk, particularly when ratios exceeded thresholds of approximately 1.4-2.0. Early work reported that ACWR values greater than 200% were associated with a 4.5-fold increase in injury risk for internal workload measures and a 3.3-fold increase for external workload measures [7]. Subsequent studies demonstrated graded increases in injury risk at lower ACWR thresholds, with ACWR values ≥42% associated with a 66% increase in injury risk [33].

Several studies also identified a protective effect of higher chronic workloads, whereby fast bowlers with well-developed chronic workload capacity demonstrated reduced injury risk despite acute workload fluctuations [33,49]. Comparative analyses showed that coupled and uncoupled ACWR calculation methods were highly correlated (R^2^ = 0.99) and yielded similar associations with injury risk, supporting methodological flexibility in ACWR computation [41].

Alternative acute-chronic exposure models, such as differential load, demonstrated comparable or superior associations with injury risk. Increases of approximately 22 overs over seven days were associated with a 2.5-fold increase in injury risk, with differential load outperforming traditional ACWR models in some analyses [43].

Evidence in Indian Fast Bowlers

Despite the breadth of literature reviewed, only one study specifically examined injury risk in Indian fast bowlers using a machine-learning approach [50]. This study reported high predictive accuracy (F1 = 0.85; area under the receiver operating characteristic curve (AUC) = 0.88) when integrating workload intensity, biomechanical variables, fitness, and injury history. No studies directly investigated Indian-specific contextual factors such as pitch characteristics, climatic conditions, match scheduling, or developmental pathways, highlighting a significant evidence gap.

Meta-Analysis

Of the 33 studies included in the systematic review, six prospective cohort studies met the predefined criteria for inclusion in the meta-analysis. These studies explicitly quantified acute workload relative to chronic workload using ACWR or conceptually aligned acute-chronic exposure models and reported extractable RR or OR estimates for injury outcomes [7,33,39,41,43,49].

**Figure 2:**
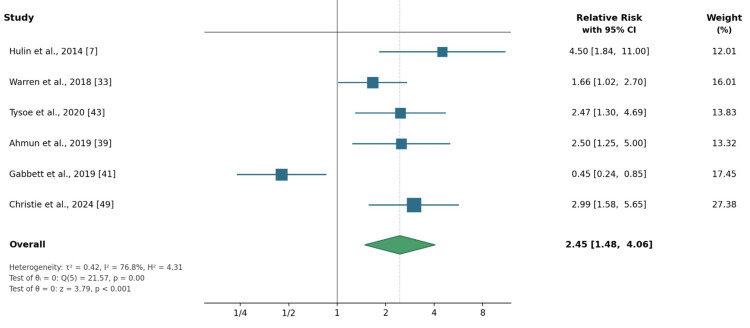
Results of random-effects meta-analysis examining the association between acute-chronic workload exposure and injury risk in cricket fast bowlers Effect estimates are reported as relative risks (RRs) with corresponding 95% confidence intervals (CIs) for each included prospective cohort study, alongside their statistical weights. The pooled analysis indicates a significantly increased injury risk associated with higher acute-chronic workload exposure (RR = 2.45; 95% CI: 1.48-4.06). Substantial between-study heterogeneity was observed (τ^2^ = 0.42; I^2^ = 76.8%; H^2^ = 4.31). The overall effect was statistically significant (z = 3.79, p < 0.001). Meta-analysis was conducted using a restricted maximum likelihood (REML) random-effects model. Studies included [7,33,43,39,41,49]

A random-effects meta-analysis using an REML estimator demonstrated a significant association between elevated acute-chronic workload exposure and injury risk, with a pooled RR of 2.45 (95% CI: 1.48-4.06). Substantial heterogeneity was observed (τ^2^ = 0.42; I^2^ = 76.8%; H^2^ = 4.31), and Cochran’s Q-test indicated significant between-study variability (Q(5) = 21.57, p < 0.001). Despite this heterogeneity, the direction of effect was consistent across all included studies, indicating a robust association between acute workload spikes relative to chronic workload and injury risk (Figure 2).

A leave-one-out sensitivity analysis was conducted to evaluate the robustness of the pooled estimate. Sequential removal of individual studies did not substantially alter the direction or significance of the pooled effect, with recalculated RR values ranging from 2.15 to 2.89 across iterations, all remaining statistically significant. Removal of Gabbett et al. (2019) [41], the only study reporting a protective effect estimate (RR = 0.45), produced the highest pooled RR (2.89; 95% CI: 2.01-4.16), while its inclusion attenuated the pooled estimate as expected. These findings support the robustness of the overall association. Formal subgroup analyses by competitive level or workload metric type were not feasible due to the limited number of included studies (n = 6).

Publication Bias

**Figure 3:**
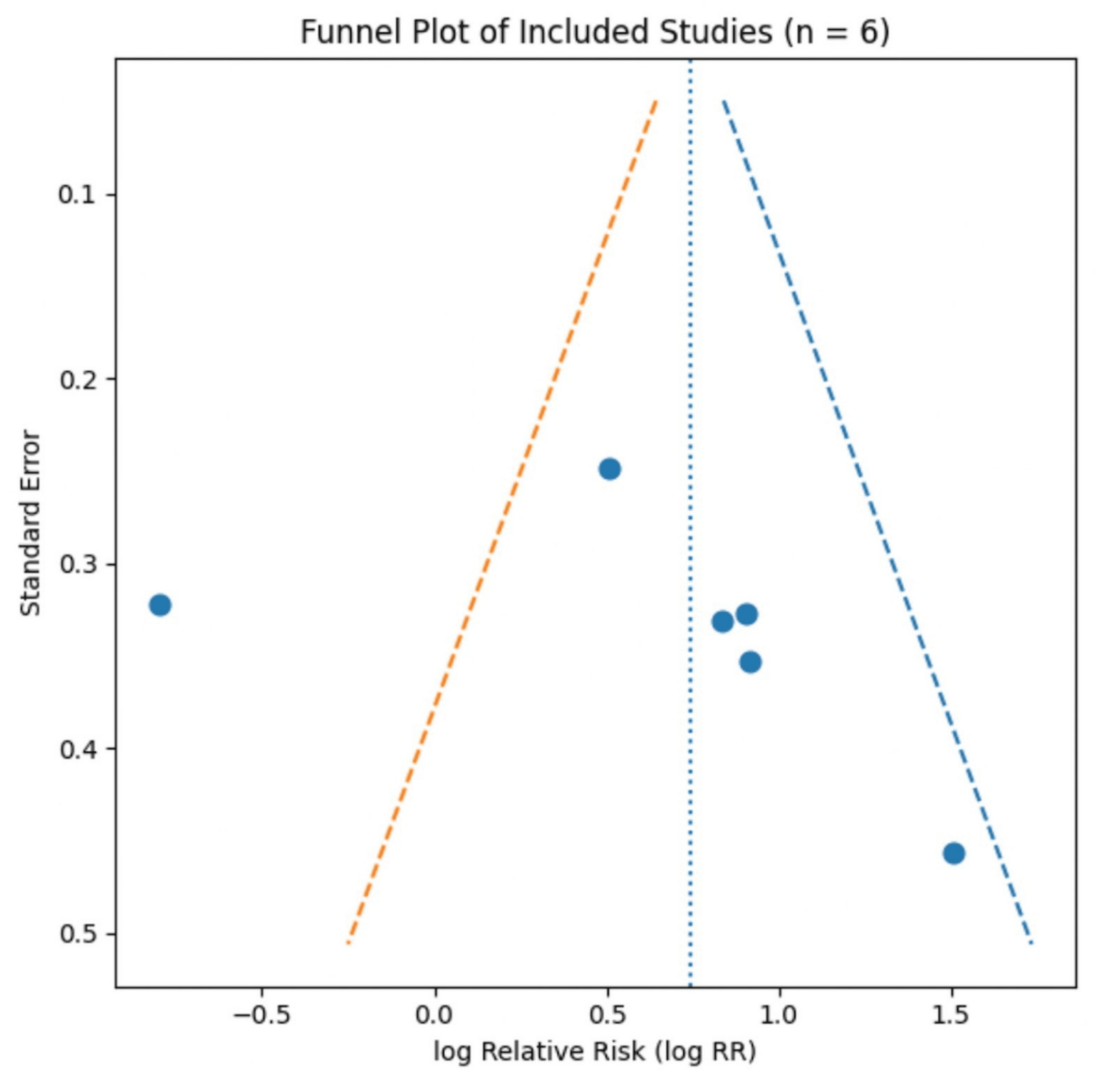
Funnel plot assessing publication bias in studies examining acute-chronic workload exposure and injury risk in cricket fast bowlers Publication bias was assessed using visual inspection of a funnel plot. The funnel plot displays the logarithm of the relative risk (log RR) against standard error for the six studies included in the meta-analysis, with each point representing an individual study. The vertical dotted line indicates the pooled effect estimate, and the diagonal dashed lines represent the pseudo 95% confidence limits. Visual inspection shows mild asymmetry, suggesting the potential presence of small-study effects or publication bias; however, given the small number of included studies (n = 6), formal statistical tests for funnel plot asymmetry were not performed, as these tests are unreliable with fewer than 10 studies. Consequently, no definitive conclusions regarding publication bias can be drawn, and interpretation should remain cautious.

Publication bias was assessed using visual inspection of a funnel plot (Figure 3). Given the small number of studies included in the meta-analysis (n = 6), formal statistical tests for funnel plot asymmetry were not performed, as these tests are unreliable with fewer than ten studies. Visual inspection did not reveal marked asymmetry; however, interpretation is limited, and no definitive conclusions regarding publication bias can be drawn.

Discussion

This systematic review and meta-analysis synthesised evidence from 33 studies examining workload quantification and injury risk in cricket fast bowlers, with a restricted quantitative synthesis of six prospective cohort studies evaluating acute-chronic workload exposure. The findings indicate a consistent association between disproportionate short-term workload relative to recent loading history and increased injury risk, while also highlighting substantial heterogeneity and methodological limitations that constrain causal interpretation.

Acute-Chronic Workload Exposure and Injury Risk

The meta-analysis identified a significant association between elevated acute workload relative to chronic workload and injury risk, with fast bowlers exposed to acute workload spikes exhibiting more than a two-fold increase in injury risk compared with those maintaining balanced workloads (pooled RR = 2.45 (95% CI: 1.48-4.06)) [7,33,39,41,43,49]. Although heterogeneity was substantial (I^2^ = 76.8%), the direction of effect was consistent across all included studies, supporting the reliability of the observed association despite variation in workload metrics, exposure windows, and athlete populations.

The observed heterogeneity (I^2^ = 76.8%) likely reflects several sources of methodological variability. These include differences in the workload metrics employed (e.g., ball counts versus sRPE-based ACWR versus differential load), inconsistencies in how injuries were defined and ascertained across studies, variation in the acute and chronic time windows used for ACWR calculation, and differences in study populations spanning junior developmental athletes to senior international fast bowlers. Additionally, contextual factors such as playing format, pitch conditions, and competition density may have contributed to between-study variability. The leave-one-out sensitivity analysis confirmed that no single study disproportionately influenced the pooled result, although removal of the Gabbett et al. (2019) [41] study, which uniquely reported a protective effect, produced the largest shift in the pooled estimate. Future individual participant data meta-analyses may help disentangle these sources of heterogeneity.

Importantly, the pooled estimate should be interpreted as associative rather than predictive or causal. Differences in workload calculation methods (e.g., ACWR, differential load), injury definitions, and analytical approaches likely contributed to between-study variability. Consequently, the findings do not support a single universal workload threshold but reinforce the principle that rapid increases in workload relative to an athlete’s recent capacity elevate injury risk.

ACWR and Alternative Acute-Chronic Models

The ACWR was the most frequently examined metric and was consistently associated with increased injury risk when thresholds exceeded approximately 1.4-2.0 [7,33]. Early work reported particularly high injury risk when ACWR exceeded 200%, with RRs up to 4.5 for internal workload measures and 3.3 for external workload measures [7]. Subsequent studies demonstrated graded increases in risk at lower thresholds, suggesting a progressive rather than binary relationship between workload spikes and injury risk [33].

Several studies also identified a protective effect of higher chronic workloads, whereby athletes with greater chronic load tolerance exhibited reduced injury risk despite acute workload fluctuations [33,49]. This supports the concept of a “chronic workload buffer” and emphasises the importance of progressive load development rather than conservative workload restriction.

Alternative acute-chronic exposure models, including differential load, demonstrated comparable or superior associations with injury risk [43]. These models may better capture the dynamic nature of load adaptation and fatigue in fast bowling, particularly in congested competition schedules. Collectively, the findings suggest that while ACWR remains a useful heuristic, no single workload metric should be interpreted in isolation.

Recovery, Bowling Frequency, and Age-Related Risk

Recovery and rest emerged as critical modifiers of workload-related injury risk. Reduced recovery between bowling sessions was strongly associated with injury, particularly in junior fast bowlers, where fewer than 3.5 rest days increased injury risk more than threefold [23]. Excessive bowling frequency and daily delivery counts were also consistently associated with overuse injuries in developing athletes [18,23].

These findings highlight the heightened vulnerability of developing athletes to workload mismanagement and underscore the need for age-appropriate workload guidelines that account for lower tissue tolerance, ongoing skeletal maturation, and less consistent access to professional monitoring in junior and sub-elite settings.

Age-specific analyses revealed distinct injury patterns across career stages. Younger fast bowlers demonstrated a higher susceptibility to bone stress injuries, including LSFs, whereas older bowlers were more prone to tendon-related injuries [16,26]. High workload and low strike rate in youth cricket strongly predicted future Test selection; multi-sport participation may enhance performance potential [30]. These findings underscore the need for age- and developmentally appropriate workload guidelines, particularly during periods of growth and transition [34].

Consistency Across Competitive Levels

The associations between high acute workloads and injury risk were broadly consistent across elite, sub-elite, and junior competitive levels. However, notable differences were observed. Elite cohorts benefited from more systematic workload monitoring infrastructure (e.g., dedicated physiotherapists, GPS-based tracking systems, athlete management databases), which may have facilitated earlier detection and management of workload spikes. In contrast, junior and sub-elite populations were comparatively more vulnerable during periods of rapid load escalation, likely reflecting less developed physical conditioning, reduced tissue tolerance, and less consistent access to professional monitoring support [23,24,28,34]. These level-specific differences suggest that while the overall workload-injury relationship applies broadly, the magnitude of risk and the effectiveness of monitoring may differ across competitive contexts.

Injury Type and Anatomical Distribution

Lumbar spine injuries were among the most prevalent and clinically significant outcomes across studies. Peak short-term workloads exceeding 234 deliveries within seven days were associated with an 11-fold increase in LSF risk [17]. Seasonal clustering of lumbar injuries during mid- and late-season competition further suggests cumulative fatigue and insufficient recovery as contributing factors.

Tissue-specific analyses indicated that bone stress injuries were associated with high medium-term workloads combined with limited career exposure, whereas tendon injuries were more closely linked to high acute and previous-season workloads [16]. Intrinsic factors such as younger age, greater height, and higher bowling speed further modified lumbar injury risk, reinforcing the multifactorial aetiology of injury in fast bowling [47].

Technology, Subjective Monitoring, and Integrated Approaches

Advances in wearable microtechnology have improved the quantification of external workload, with measures such as PlayerLoad™ demonstrating strong correlations with perceived effort and bowling intensity [29,37]. Technology-informed interventions, such as modified approach lengths, have also demonstrated meaningful reductions in lumbar loading with minimal performance compromise [37].

Internal workload measures, particularly sRPE, were widely used and associated with injury risk when short-term loads were elevated [39,41]. However, recall bias and reduced accuracy with delayed reporting remain limitations [48]. Evidence suggests that integrating subjective and objective measures provides a more comprehensive representation of injury risk than either approach alone.

Evidence Gaps and the Indian Context

Despite India’s prominence in global cricket, only one included study directly examined injury risk in Indian fast bowlers [50]. As a result, conclusions specific to Indian fast bowlers cannot be drawn from the current evidence base. Instead, this review identifies Indian fast bowling as a significant research gap, particularly given unique contextual factors such as dense competition schedules, varied pitch conditions, climatic extremes, and heterogeneous developmental pathways.

Future research should prioritise region-specific longitudinal studies to determine whether workload-injury relationships observed in predominantly Australian and English cohorts generalise to Indian fast bowlers.

Methodological Considerations

The primary limitation of this review lies in the heterogeneity of included studies, which varied in design, workload metrics, injury definitions, and statistical reporting. Although a restricted meta-analysis was feasible for acute-chronic workload exposure, many studies lacked sufficient statistical detail for quantitative synthesis. Additionally, female fast bowlers remain underrepresented, limiting sex-specific inference.

The small number of studies included in the meta-analysis (n = 6) precluded formal subgroup analyses by competitive level, workload metric type, or sex. Although the leave-one-out sensitivity analysis supported the robustness of the pooled estimate, future meta-analyses with a larger pool of eligible studies should incorporate subgroup and meta-regression analyses to explore sources of heterogeneity more systematically.

## Conclusions

This systematic review and meta-analysis indicate that acute workload spikes relative to recent chronic workload are consistently associated with increased injury risk in cricket fast bowlers. While the magnitude of risk varies across studies, the direction of effect is consistent, supporting the use of workload monitoring as a risk management tool rather than a deterministic injury prediction model.

The findings emphasise the importance of gradual workload progression, adequate recovery, and individualised monitoring across age groups and competitive levels. Although ACWR remains a commonly used metric, alternative acute-chronic exposure models may offer improved sensitivity in certain contexts. Crucially, the limited evidence specific to Indian fast bowlers highlights the need for context-aware, longitudinal research to inform workload guidelines tailored to regional demands. Overall, workload management in fast bowling should be guided by integrated, athlete-centred frameworks that combine objective workload data, subjective monitoring, and clinical judgement to reduce injury risk while supporting performance development.
